# Construction and analysis of a lncRNA–miRNA–mRNA competing endogenous RNA network from inflamed and normal synovial tissues after anterior cruciate ligament and/or meniscus injuries

**DOI:** 10.3389/fgene.2022.983020

**Published:** 2022-10-17

**Authors:** Xiling Xiao, Xiaofan Yang, Sen Ren, Chunqing Meng, Zhaohui Yang

**Affiliations:** ^1^ Department of Rehabilitation, Union Hospital, Tongji Medical College, Huazhong University of Science and Technology, Wuhan, China; ^2^ Department of Hand Surgery, Union Hospital, Tongji Medical College, Huazhong University of Science and Technology, Wuhan, China; ^3^ Department of Neurosurgery, Zhongnan Hospital of Wuhan University, Wuhan, China; ^4^ Department of Orthopaedics, Union Hospital, Tongji Medical College, Huazhong University of Science and Technology, Wuhan, China

**Keywords:** anterior cruciate ligament, meniscus, synovitis, RNA sequencing, competing endogenous RNA, immune infiltration

## Abstract

**Background:** Despite ample evidence demonstrating that anterior cruciate ligament (ACL) and meniscus tears are associated with posttraumatic osteoarthritis (PTOA) development, the contributing factors remain unknown. Synovial inflammation has recently been recognized as a pivotal factor in the pathogenesis of OA. However, there is a lack of data on synovial profiles after ACL or meniscus injuries, which may contribute to PTOA.

**Methods:** Twelve patients with ACL tears and/or meniscus injuries were recruited. During surgery, synovial tissues were obtained from the injured knees. The inflammation status of the synovium was characterized according to macroscopic criteria and histological synovitis grades. Then the synovial tissues were classified as control group or inflamed group. High-throughput RNA sequencing of the synovial samples (3 vs. 3) was conducted to identify differentially expressed (DE) RNAs. Gene Ontology (GO), Kyoto Encyclopedia of Genes and Genomes (KEGG) pathway, and protein–protein interaction (PPI) analyses were performed to investigate DE mRNAs. Next, competing endogenous RNA (ceRNA) networks were constructed based on bioinformatics analyses. Associations of the identified DE genes (DEGs) with infiltrating immune cells were explored using Pearson correlation analysis.

**Results:** The results showed that 2793 mRNAs, 3392 lncRNAs and 211 miRNAs were significantly DE between two groups. The top 3 significantly upregulated GO terms and KEGG pathways were immune response, adaptive immune response and immune system process, systemic lupus erythematosus, haematopoietic cell lineage and cytokine–cytokine receptor interaction, respectively. In PPI networks, the top 10 hub genes were IL6, CCR7, C3, CCR5, CXCR3, CXCL8, IL2, CCR3, CCR2 and CXCL1. Seven mRNAs (EPHA5, GSN, ORC1, TLN2, SOX6, NKD2 and ADAMTS19), 4 lncRNAs (MIR4435-2HG, TNXA, CEROX1 and TMEM92-AS1) and 3 miRNAs (miR-486-5p, miR-199a-3p and miR-21-3p) were validated by quantitative real-time polymerase chain reaction and sub-networks were constructed. In correlation analysis, MMP9 correlated positively with M0 macrophages and plasma cells, NKD2 positively with CD8 T cells, and CCR7 and IL2RB positively with naive B cells.

**Conclusion:** Our study provides foundational synovial inflammation profiles following knee trauma. The ceRNA and PPI networks provide new insight into the biological processes and underlying mechanisms of PTOA. The differential infiltration profiles of immune cells in synovium may contribute to PTOA development. This study also highlights immune-related DEGs as potential PTOA treatment biomarkers.

## Introduction

Osteoarthritis (OA) is the most prevalent form of joint diseases and a major health burden. It is predicted to be the leading cause of disability in the general population by 2030 (Thomas et al., 2014). Traumatic meniscal and anterior cruciate ligament (ACL) tears are major causes of this degenerative disease. Astoundingly, approximately 50% of patients eventually develop posttraumatic OA (PTOA) within 10–20 years after joint injury (Lohmander et al., 2007; Oiestad et al., 2009).

However, the mechanism that contributes to the development of PTOA is still unknown. Several studies have detected alterations in knee kinematics and kinetics following ACL tears and meniscus injuries (Akpinar et al., 2018; Kotsifaki et al., 2020; Wang et al., 2021), which may contribute to both initiation and progression of OA development. Recently, a renewed focus has been placed on the inflammatory milieu after acute traumatic injury. After joint trauma, increased levels of proinflammatory cytokines, catabolic enzymes, and biomarkers of cartilage breakdown and bone turnover have been detected in synovial fluid, blood, and urine (Lohmander et al., 2003; Alonso et al., 2020; Hagemans et al., 2021; Lisee et al., 2021), which may also play a role in PTOA development. Moreover, a subset of patients after ACL injury who demonstrate significantly greater concentrations of biomarkers show more severe PTOA progression (Amano et al., 2018; Jacobs et al., 2020). Notably, patients show variability in responses to anti-inflammatory treatment after ACL injury (Lattermann et al., 2017), underscoring the need to determine whether different ACL patient phenotypes exist and to explore potential therapeutic targets to lessen chondral degeneration following ACL injury. Recently, proteomic analyses have been performed on synovial fluid collected from patients after acute ACL injury (King et al., 2020). In addition, the immune cell profiles of the joint synovial fluid from individuals with ACL or meniscus injuries have been evaluated (Kim-Wang et al., 2021). However, the synovial membrane profiles that contribute to or mediate OA progressive cartilage degradation and joint dysfunction have not been studied in patients with ACL and meniscus injuries (Ayral et al., 2005; Sellam and Berenbaum, 2010; Robinson et al., 2016).

Only a few studies have evaluated synovial biopsy characterizations that are altered following joint injury in animal models. Specifically, recent animal model experiments have shown that the synovium may be involved in the progression of PTOA after anterior cruciate ligament rupture or medial meniscus transection (Zhou et al., 2018; Salazar-Noratto et al., 2019; Kuroki et al., 2021). In addition, several studies have evaluated synovial profiles in patients with OA (Xiang et al., 2019a; Xiang et al., 2019b; Zhou et al., 2020), showing that noncoding RNAs may play key roles in OA synovitis and may have prospective importance in OA diagnosis and therapy. Furthermore, different gene expression patterns between inflamed and normal areas of the synovium could reveal key pathways involved in OA pathogenesis and provide new potential targets of treatment (Lambert et al., 2014). Future studies investigating molecular mechanisms and biologic pathways in synovial membrane may provide an enhanced understanding of the pathophysiology and development of PTOA following joint injury. However, to the best of our knowledge, there is a lack of data on synovial profiles in patients after ACL or meniscus injuries. In addition, studies have demonstrated that noncoding RNA expression patterns are potentially disease specific (Tavallaee et al., 2020; Wang et al., 2020), supporting the need for further studies focused on profiling noncoding RNAs in the contexts of ACL/meniscus injuries.

Therefore, in this study, high-throughput RNA sequencing (RNA-seq) was conducted to systematically explore different expression profiles of microRNAs (miRNAs), mRNAs and long noncoding RNAs (lncRNAs) between inflamed and normal synovial tissues from patients undergoing ACL and/or meniscus injuries. Moreover, lncRNA–miRNA–mRNA competing endogenous RNA (ceRNA) and protein–protein interaction (PPI) networks were established. Therefore, the purpose of our study was to provide greater insights into molecular mechanisms and biologic pathways that could regulate PTOA progression. In addition, this study aimed to identify new potential targets for the prevention and earlier treatment of PTOA following ACL/meniscus injuries.

## Materials and methods

### Patients and specimens

Synovial tissue samples from 12 patients with knee trauma were obtained at the time of ACL reconstruction and/or meniscus repair or meniscectomy at the Department of Orthopaedics, Union Hospital, Tongji Medical College, Huazhong University of Science and Technology. This study was approved by the Ethics Committee at Tongji Medical College (IORG No. IORG0003571), and all subjects provided informed consent.

Patients between 18 and 45 years of age who had a traumatic knee injury and were scheduled for arthroscopic knee procedures were recruited from the Department of Orthopaedics, Union Hospital. The inclusion criteria existence of an isolated ACL injury as determined *via* clinical examination (positive Lachman test) and validated using magnetic resonance imaging examination and/or a meniscal tear identified on preoperative MRI corresponding to the clinical presentation. The exclusion criteria for the current study included previous traumatic injury to the affected knee, history of diagnosed arthritis, and systemic inflammatory or autoimmune disease.

All the synovium were collected from suprapatellar bursa in all patients and by the same surgeon using biopsy forceps. The inflammation status of the synovial membrane was characterized by the surgeon according to macroscopic criteria (Ayral, 1996). The synovial membrane was classified into 3 grades, normal, reactive, and inflamed, according to the criteria established by Ayral on the basis of synovial vascularization, villus formation, and hypertrophy of the tissue. The synovial samples were divided into two groups for this study: the normal (control) group and the inflamed group. Twelve individuals (Table 1) met the inclusion criteria. Normal or inflamed synovial biopsy samples were immediately submerged in RNAlater™ Stabilization Solution (Thermo Fisher Scientific, Vilnius, Lithuania). Each sample was stored at 4°C overnight followed by −80°C until RNA extraction.

**TABLE 1:** Characteristics and clinical findings of patient groups.

Subject	Sex	Age (years)	BMI (kg/m^2^)	Time from injury to surgery (days)	Injury type (ACL/meniscal tear)	Status of synovium (inflamed/normal)	Articular cartilage (normal/degenerative/defects)
I1	M	30	25.71	14	ACL and medial meniscal tear	Inflamed	Normal
I2	F	31	28.28	14	ACL tear	Inflamed	Normal
I3	F	43	19.92	21	ACL and medial meniscal tear	Inflamed	Normal
I4	M	21	22.49	30	ACL tear	Inflamed	Normal
I5	M	30	28.4	21	lateral meniscal tear	Inflamed	Normal
I6	F	44	25.39	20	medial meniscal tear	Inflamed	Normal
C1	M	41	27.68	14	ACL and medial meniscal tear	Normal	Normal
C2	M	23	29.24	30	ACL and meniscal tear	Normal	Normal
C3	M	31	31.52	27	ACL, medial and lateral meniscal tear	Normal	Normal
C4	M	31	24.22	60	ACL tear	Normal	Normal
C5	M	27	19.38	60	ACL and medial meniscal tear	Normal	Normal
C6	F	22	20.51	24	lateral meniscal tear	Normal	Normal

I1-I6 indicated subjects in the inflamed group and C1–C6 represented subjects in the control group.

### Hematoxylin-eosin staining

At the same time, the histological features of the collected synovium samples were verified by hematoxylin-eosin (H&E) staining. All the synovial biopsy samples were fixed, dehydrated in xylene, and embedded in paraffin according to standard procedures. Five-micrometer sections were cut and deparaffinized using a standard protocol. These sections were stained with hematoxylin and eosin (H&E) to confirm the inflammatory status of the synovial samples.

### RNA extraction and RNA-seq analysis

Total RNA was extracted from the synovial tissues using TRIzol (Invitrogen, Carlsbad, CA, United States) according to the manufacturer’s protocol. The RNA integrity and concentration were assessed using a Nano Drop and an Agilent 2100 bioanalyzer (Thermo Fisher Scientific, MA, United States). Appropriate quality of RNAs were stored at −80°C for mRNA, lncRNA and miRNA sequencing and subsequent experiments.

After the total RNA from each sample was qualified, RNA-seq was performed to identify the differentially expressed (DE) mRNAs, lncRNAs and miRNAs between both groups. Three inflamed subjects and three control subjects were chosen randomly for microarray analysis. Standard cDNA libraries were constructed and sequenced using the DNBSEQ platform (BGI-Shenzhen, China). Furthermore, differential expression analysis was performed using DEGseq (Wang et al., 2010). The significantly dysregulated RNAs had to meet the following criteria: fold changes ≥ 1.5 or ≤ −1.5, *p* and *q* values < 0.05.

### Gene ontology and kyoto encyclopedia of genes and genomes enrichment analyses

The differentially expressed mRNAs were analyzed with the Gene Ontology (GO) database and Kyoto Encyclopedia of Genes and Genomes (KEGG) pathway database. The GO categories (http://geneontology.org) were used to define the molecular mechanisms and biological functions of the candidate genes. The biological functions of these genes were further annotated with the KEGG database (http://www.genome.jp/kegg). The hypergeometric distribution test was used to identify significantly enriched gene sets. A *p* value < 0.05 was considered to indicate statistical significance.

### Real-time polymerase chain reaction

The remaining RNAs of synovial samples in each group (*n* = 3 per group) were used to validate the RNA-seq results by using real-time polymerase chain reaction (RT–PCR). miRNAs and other RNAs were reverse-transcribed into cDNA using iTaq™ M-MLV reverse transcriptase (#M170A, Promega) and an iScript cDNA Synthesis Kit (#1708890, Bio–Rad) according to the respective user manuals. RT–PCR was performed on a CFX Connect platform (Bio–Rad, United States) using iTaq™ Universal SYBR Green Supermix (#1725124, Bio–Rad). The primer sequences are presented in Supplementary Tables S1–S3. The relative expression levels of targeted genes were calculated using the 2^−ΔΔCt^ method, and the data were normalized to gapdh (endogenous internal control for mRNA and lncRNA) or u6 (endogenous internal control for miRNA).

### Construction of the competing endogenous RNA and protein–protein interaction network

The significantly DE mRNAs and lncRNAs between both groups were used for ceRNA network construction. We used the targeting relationships of mRNAs/lncRNAs regulated by miRNAs to establish a lncRNA–miRNA–mRNA interaction network. The RNAs that could be predicted by at least two of the databases [RNAhybrid (https://bibiserv.cebitec.uni-bielefeld.de/rnahybrid), miRanda (http://www.microrna.org/microrna/home.do) and TargetScan (http://www.targetscan.org)] were considered miRNA targets. The sequences of mRNAs and lncRNAs were screened to obtain the potential miRNA response elements. Protein–protein interaction (PPI) analysis of the DE mRNAs was performed based on the STRING database (https://string-db.org). These networks were illustrated using Cytoscape 3.7.1. The degree centrality of the involved genes was calculated by Cytoscan.

### Construction of the competing endogenous RNA and protein–protein interaction network

The significantly DE mRNAs and lncRNAs between both groups were used for ceRNA network construction. We used the targeting relationships of mRNAs/lncRNAs regulated by miRNAs to establish a lncRNA–miRNA–mRNA interaction network. The RNAs that could be predicted by at least two of the databases [RNAhybrid (https://bibiserv.cebitec.uni-bielefeld.de/rnahybrid), miRanda (http://www.microrna.org/microrna/home.do) and TargetScan (http://www.targetscan.org)] were considered miRNA targets. The sequences of mRNAs and lncRNAs were screened to obtain the potential miRNA response elements. Protein–protein interaction (PPI) analysis of the DE mRNAs was performed based on the STRING database (https://string-db.org). These networks were illustrated using Cytoscape 3.7.1. The degree centrality of the involved genes was calculated by Cytoscan.

### Correlation analysis between DEGs and immune cells

**FIGURE 1:**
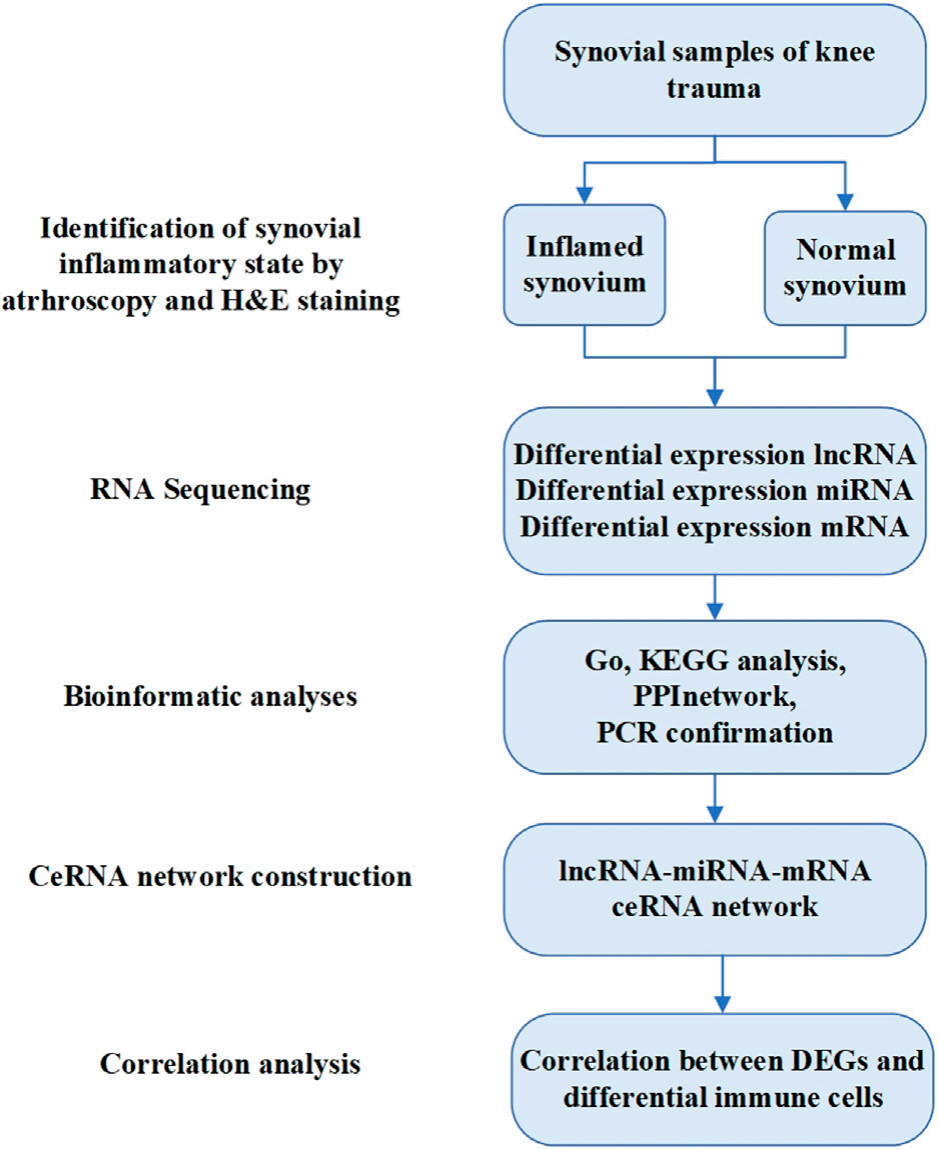
The workflow diagram of this study.

The association between the identified DEGs and levels of infiltrating immune cells was explored using Pearson correlation analysis in R software. The resulting associations were visualized using the chart technique with the “ggplot2” package. The complete workflow is shown in Figure 1.

### Statistical analysis

Statistical analysis was calculated with GraphPad Prism v 8.3.0 software. All data were expressed as the mean ± SEM. Unpaired Student’s *t* test was performed for comparisons between two groups. Pearson correlation analysis was used to assess the constructed ceRNA network. Statistical significance was set at *p* or *q* < 0.05.

## Results

### Histological features of synovial biopsy samples

**FIGURE 2:**
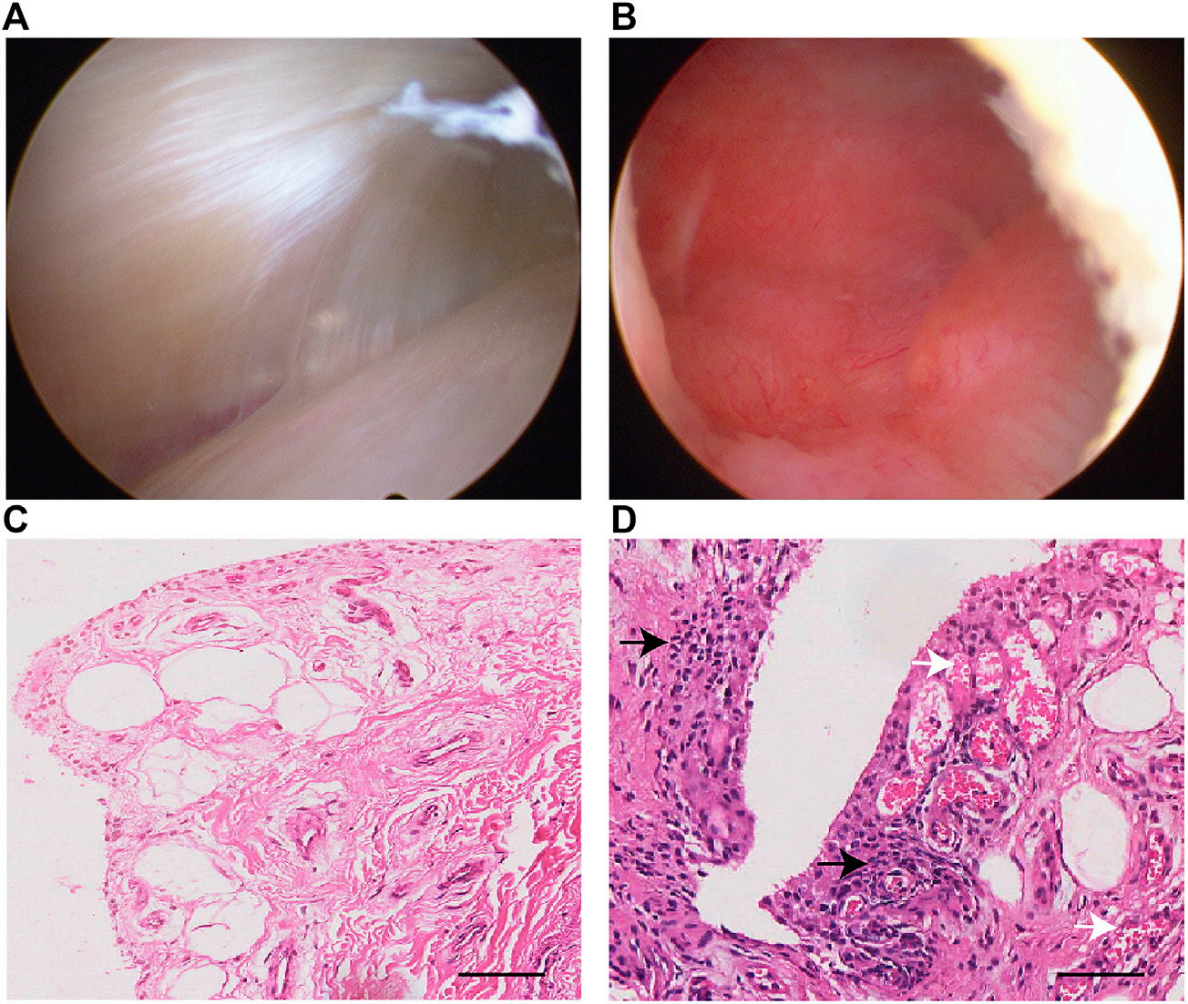
Representative arthroscopy view and H&E staining of normal **(A,C)** and inflamed **(B,D)** synovial samples in this study. The synovial inflammatory state may result in a pannus-like response covering the suprapatellar bursa **(B)**. Inflammatory cells infiltration in the synovial lining and sublining is seen (black arrow), along with vascular proliferation (white arrow) in subintimal layer **(D)**. Scale bar 50 μm.

Twelve samples from subjects with ACL and/or meniscus injuries were collected (Table 1). During each arthroscopic knee procedure, we collected synovial tissue at the time of surgery for laboratory analysis. The gross appearances of synovial membranes assessed by the surgeon during arthroscopy were confirmed by the histological characteristics. The histological features were highly consistent with the macroscopic views as shown in Figure 2.

### Differential expression analyses

**FIGURE 3:**
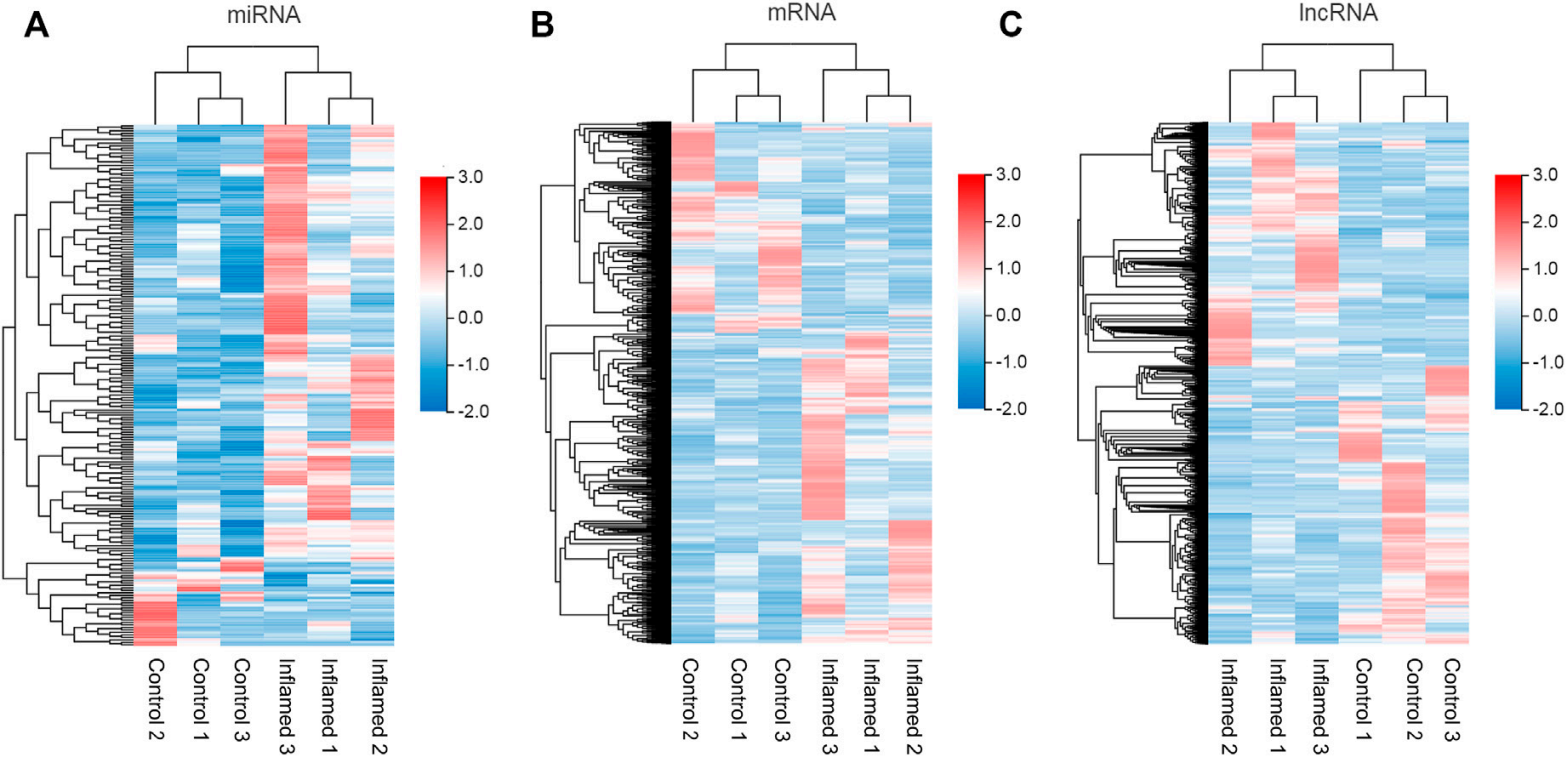
Expression profiles of RNAs. **(A–C)** the miRNAs, mRNAs and lncRNAs, profiles were shown in heatmap in synovium from inflamed group and control group. *N* = 3.

To identify functional mRNAs, lncRNAs, and miRNAs involved in the inflammatory mechanisms of the synovial membrane, total RNA of synovial tissue samples randomly selected from the inflamed groups and control groups (*n* = 3) was collected for RNA-seq. All the DE miRNAs, mRNAs and lncRNAs are presented in the hierarchical clustering heatmaps in Figure 3.

In total, 211 differentially expressed miRNAs (177 upregulated and 34 downregulated), 2793 differentially expressed mRNAs (1737 upregulated and 1056 downregulated) and 3392 differentially expressed lncRNAs (1623 upregulated and 1769 downregulated) were detected in the inflamed group compared with the control group (Figures 3A–C; Supplementary Data S1–S3).

### Functional enrichment analysis of differentially expressed mRNAs

**FIGURE 4:**
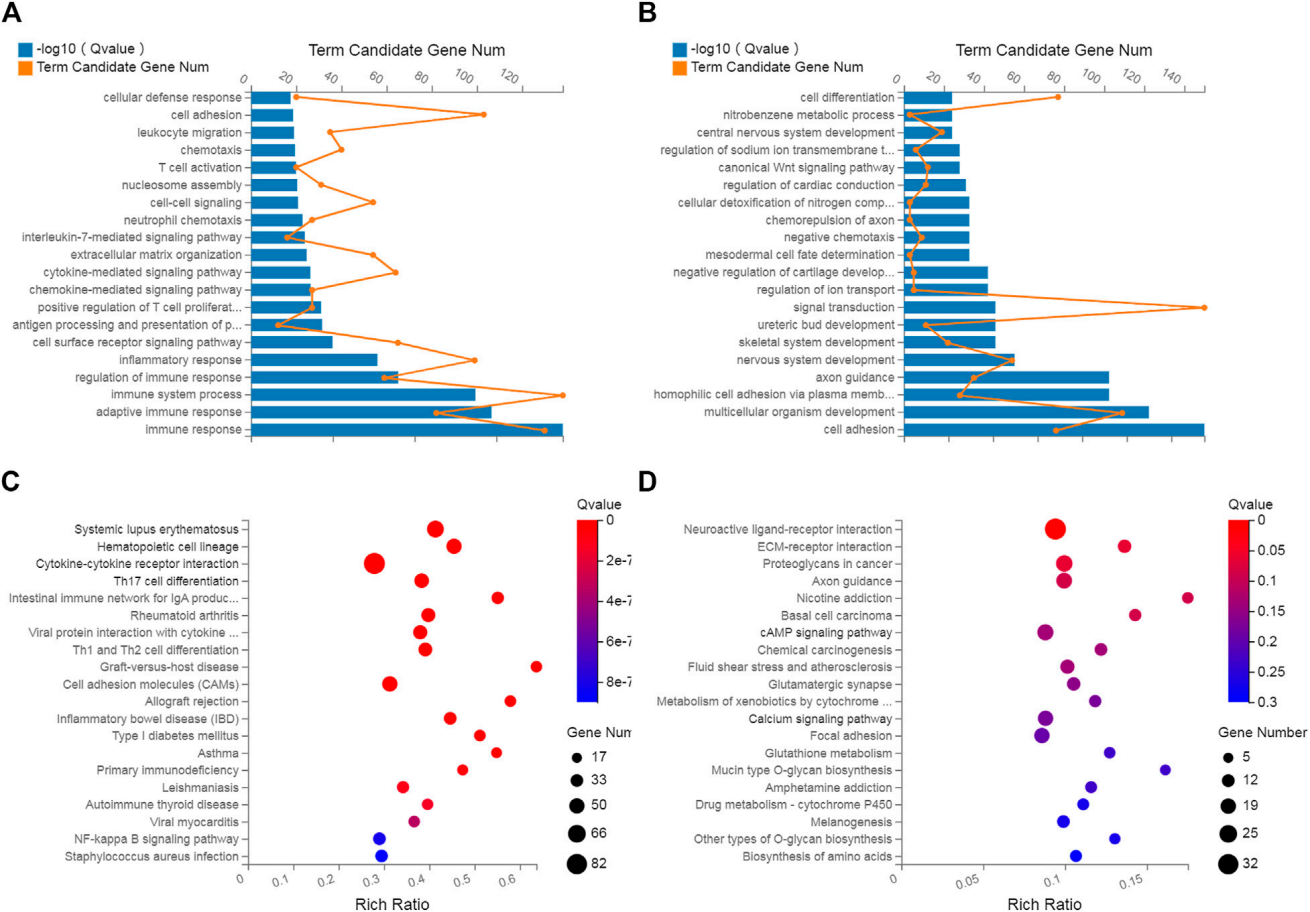
GO analysis and KEGG pathway analysis. GO annotation of biological processes related to upregulated and downregulated mRNAs **(A,B)**, KEGG pathway enrichment analysis of upregulated and downregulated mRNAs **(C,D)** in inflamed synovium compared with normal synovium.

GO and KEGG analyses were performed to investigate the biological effects of the dysregulated mRNAs. First, GO analyses were performed to analyze the up- and downregulated differentially expressed targeted genes. The results revealed that 5055 upregulated and 3571 downregulated GO terms were enriched in the biological process (BP) category. Moreover, 136 GO terms were significantly upregulated, and 15 GO terms were significantly downregulated (Supplementary Data S4, S5). The top upregulated and downregulated enriched terms are listed in Figures 4A,B. Several significant GO terms, such as immune response, immune system process, inflammatory response and chemotaxis, were potentially associated with inflammatory and immune processes following ACL/meniscus injuries (Figure 4A).

**FIGURE 5:**
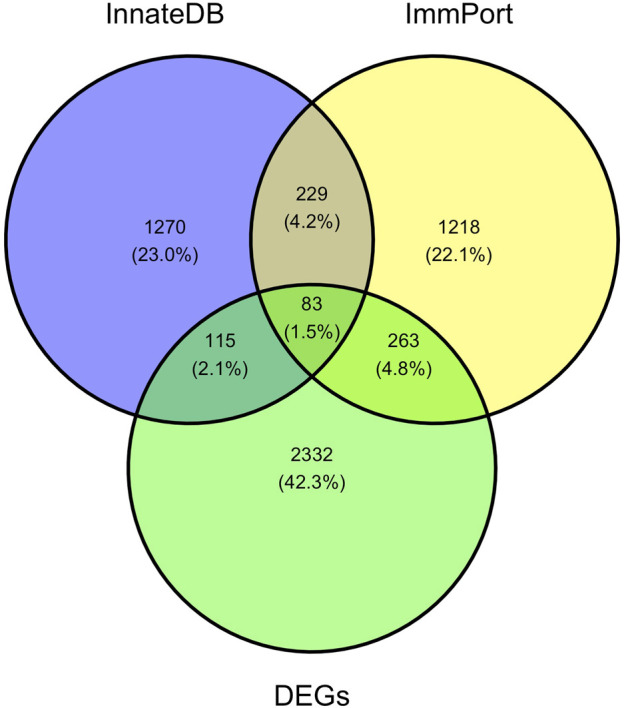
A Venn diagram of DEGs, immunerelated genes in InnateDB and Immport databases. A total of 83 DEGs are obtained through the intersection of the immune-related DEGs identified in this study, InnateDB and Immport database.

Pathway enrichment analyses were used to explore key signaling pathways of PTOA. The results showed 41 upregulated and 1 downregulated markedly enriched pathways, as shown in Supplementary Data S6, S7. The top upregulated and downregulated enriched pathways are listed in Figures 4C,D. Among the top upregulated signaling pathways were immune response and inflammatory pathways, such as the rheumatoid arthritis (RA), Th17-cell differentiation, hematopoietic cell lineage and cytokine–cytokine receptor interaction pathways. In comparison, two immune gene databases (InnateDB and Immport) were selected to overlap the DEGs with immune genes. A Venn diagram was constructed, as shown in Figure 5. Eight-three overlapping genes are listed in Supplementary Data S8.

### Protein–protein interaction network construction

**FIGURE 6:**
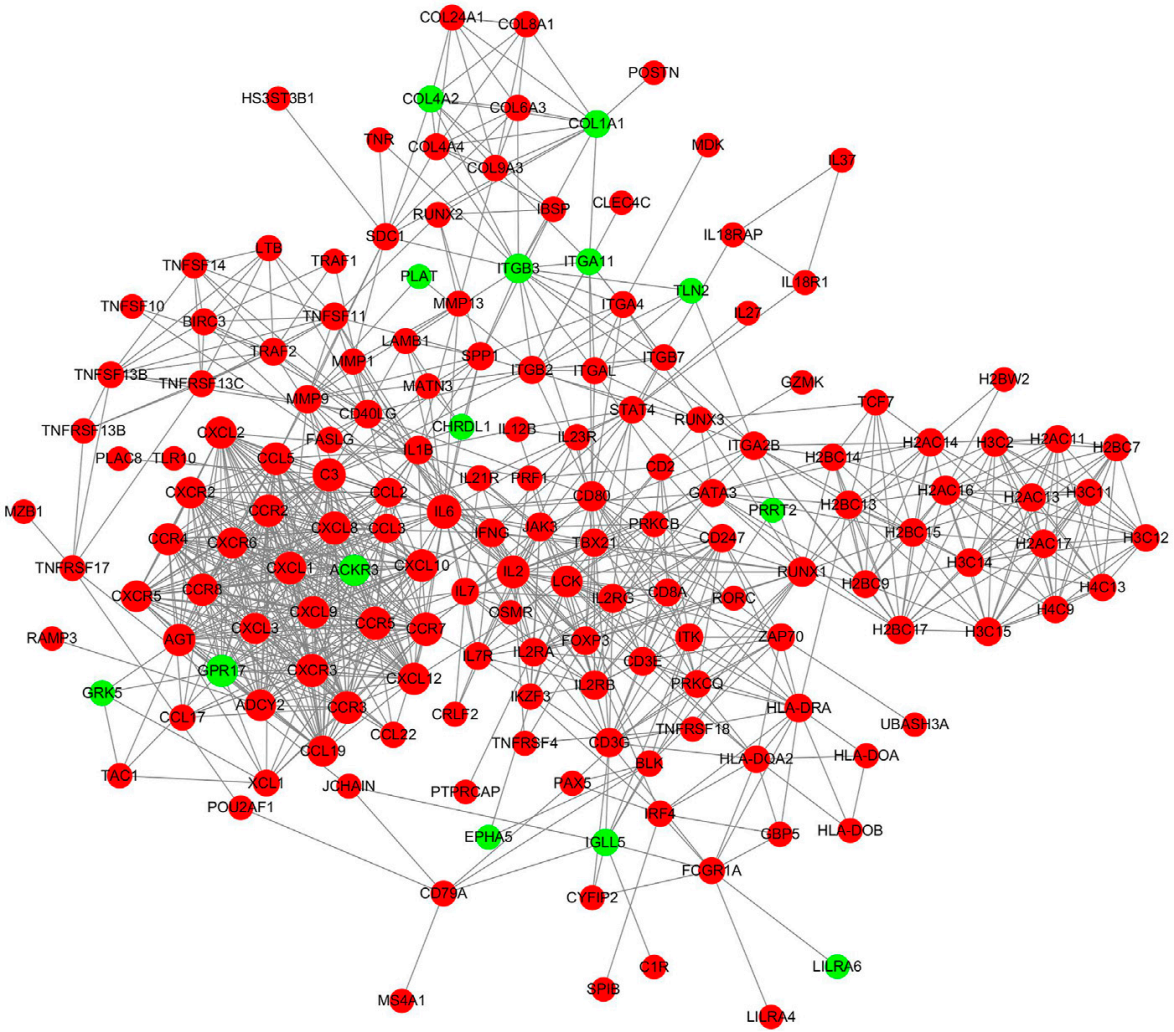
PPI network analyses of the differentially expressed mRNAs. The red nodes represented upregulated mRNAs and green nodes represented downregulated mRNAs.

PPI networks were constructed to identify critical genes among the differentially expressed mRNAs. When the inflamed group was compared with the control group, the established network comprised 164 nodes and 923 edges (Figure 6). The topological characteristics of nodes in the PPI network are displayed in Supplementary Data S9. In this network, the top 15 genes with the highest core degrees were IL6, CCR7, C3, CCR5, CXCR3, CXCL8, IL2, CCR3, CCR2, CXCL1, CXCL10, CCL5, AGT, CCR8 and CXCL2.

### Validation of differentially expressed microRNAs

**FIGURE 7:**
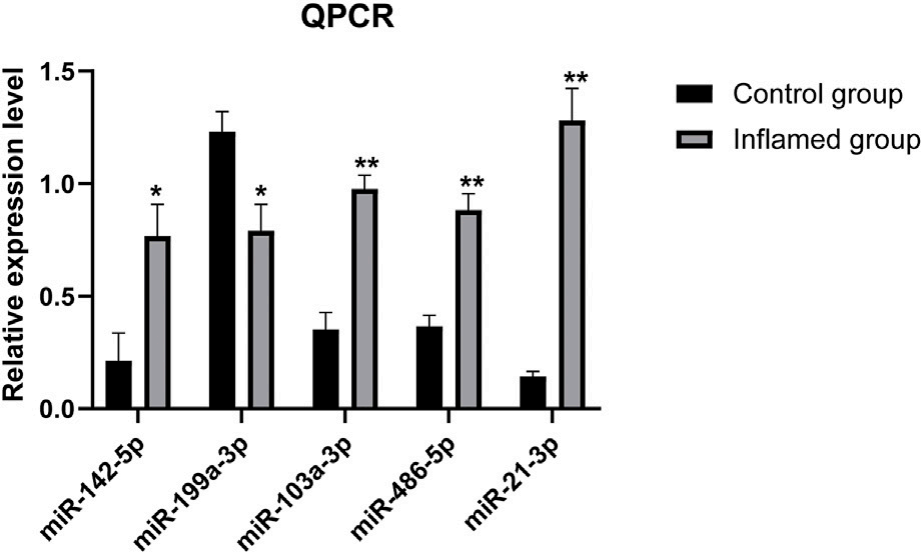
PCR analysis of the differentially expressed miRNAs. *N* = 3. ∗*p* < 0.05, ∗∗*p* < 0.01.

RT–PCR analysis was performed on RNA extracted from synovial tissues to confirm the expression level changes gained from the RNA-seq analysis. We randomly selected 11 miRNAs with high fold change to validate the reliability of the RNA-seq data. PCR analysis verified that miR-21-3p, miR-486-5p, miR-142-5p and miR-199a-3p were upregulated/downregulated in the inflamed group compared with the control group. These results were consistent with the RNA-seq data (Figure 7). However, PCR analysis showed that miR-103a-3p was upregulated in the inflamed group, which conflicted with the RNA-seq data. The expressions of the remaining six miRNAs were not significantly different between groups according to PCR analysis.

### Construction of the lncRNA–miRNA–mRNA network

**FIGURE 8:**
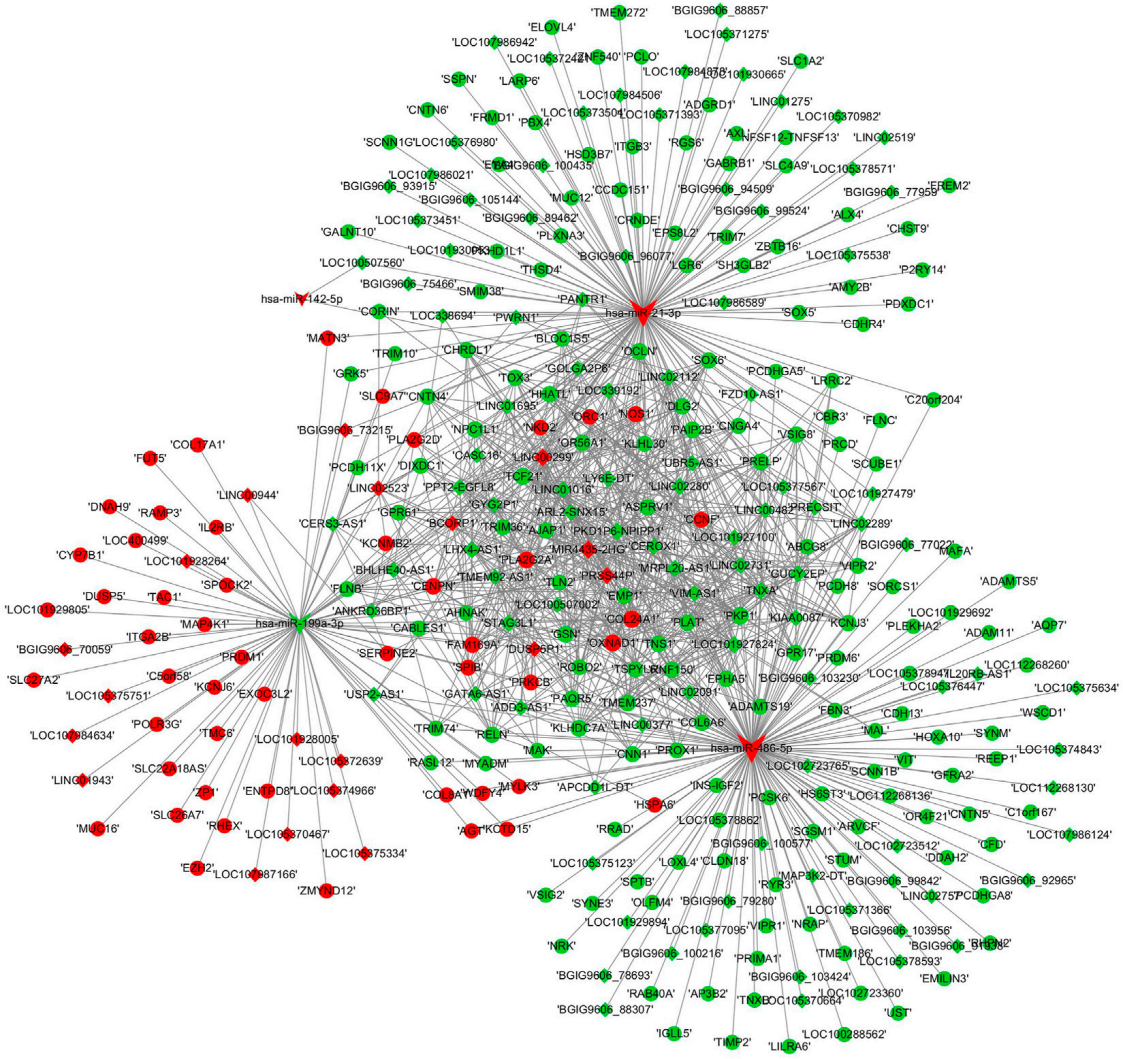
ceRNA network construction. lncRNA–miRNA–mRNA networks were established based on the differentially expressed lncRNAs, mRNAs, and validated miRNAs. miRNAs were indicated to V-shape, lncRNAs were indicated to rhombus and mRNAs were indicated to ellipse. The red nodes represented upregulated RNAs and green nodes represented downregulated RNAs. The size of a node indicated the degree of node in the network.

A ceRNA regulatory network was established based on the differentially expressed RNAs. miRNAs validated by RT–PCR analysis, which also showed expression patterns consistent with the RNA-seq results, were selected as the cores of the ceRNA network. The lncRNA–miRNA–mRNA ceRNA network comprised four miRNAs, 211 mRNAs, and 120 lncRNAs (Figure 8). The topological characteristics of nodes in the ceRNA network are displayed in Supplementary Data S10.

### Real-time polymerase chain reaction validation of differentially expressed mRNAs and long noncoding RNAs

**FIGURE 9:**
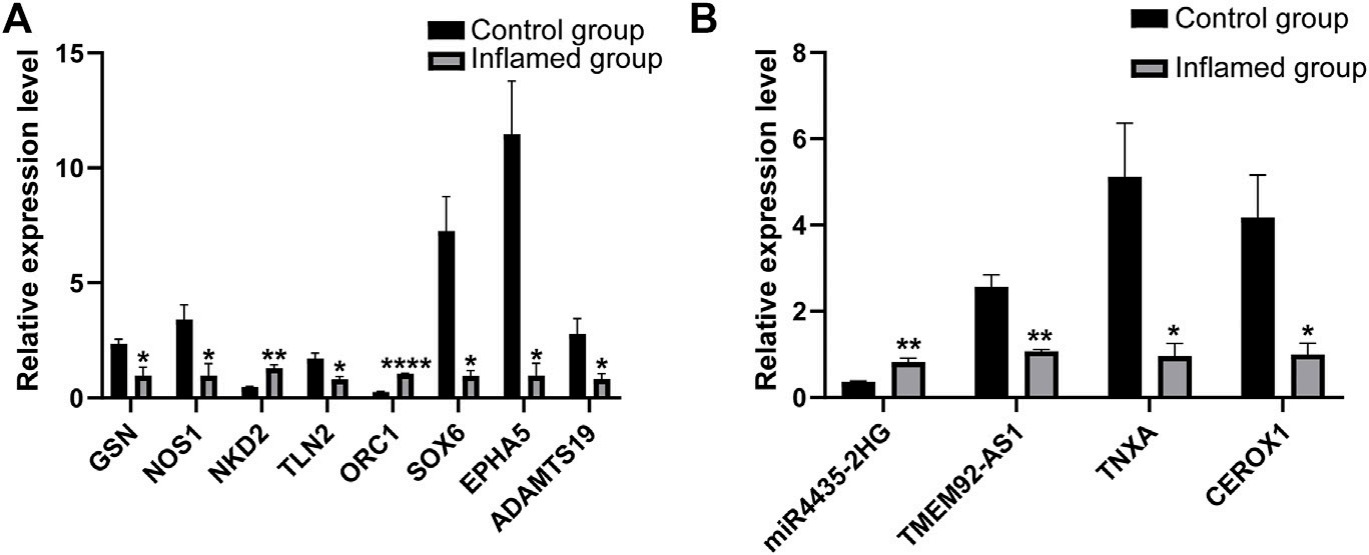
PCR analysis of the differentially expressed mRNAs **(A)** and lncRNAs **(B)**. *N* = 3. *p < 0.05, **p < 0.01, ***p < 0.001, ****p < 0.0001.

Further RT–PCR analysis of 14 mRNAs and 8 lncRNAs included in the ceRNA networks was performed to confirm the RNA-seq results. The RT–PCR results showed that seven mRNAs were significantly dysregulated (Figure 9A). These results were found to be consistent with the RNA-seq data. However, PCR analysis showed that one mRNA (NOS1) was downregulated in the inflamed group, which conflicted with the RNA-seq data. The expression of the remaining six mRNAs was not significantly different between the groups according to PCR analysis.

Among the dysregulated mRNAs, GSN, ORC1, SOX6, NKD2 and ADAMTS19 were involved in the ceRNA network (Figure 8); EPHA5 and TLN2 were involved in both the PPI and ceRNA networks (Figures 6, 8). Moreover, three lncRNAs (TMEM92-AS1, TNXA and CEROX1) were significantly downregulated, while one lncRNA (MIR4435-2HG) was significantly upregulated in the inflamed group compared with the control group (Figure 9B). The expression of the remaining four lncRNAs did not significantly differ between the groups according to PCR analysis.

### Establishment of a competing endogenous RNA subnetwork based on RNAs verified by polymerase chain reaction analyses

**FIGURE 10:**
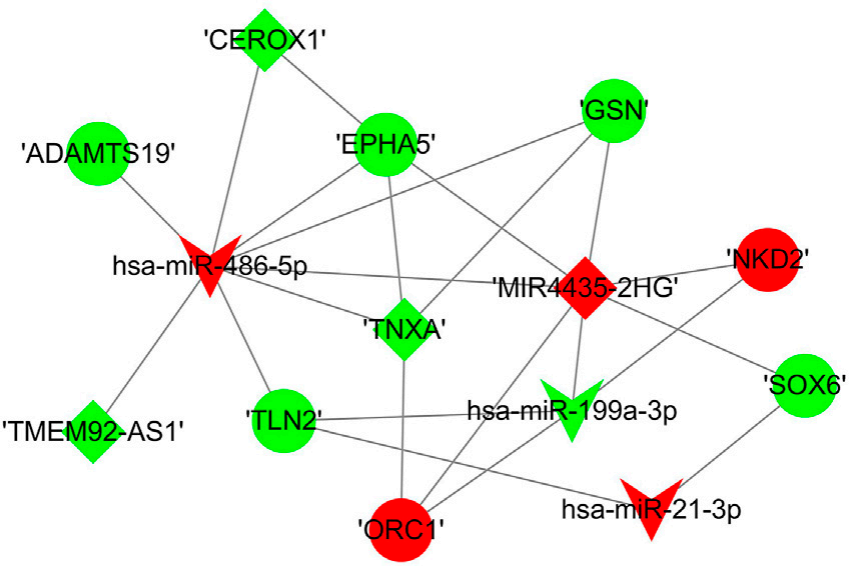
The sub competing endogenous RNA network. miRNA were indicated to V-shape, lncRNAs were indicated to rhombus and mRNAs were indicated to ellipse. The red nodes represented upregulated RNAs and green nodes represented downregulated RNAs. The size of a node indicated the degree of node in the network.

We constructed a lncRNA-miRNA-mRNA subnetwork using the above validated PCR results for further investigation. The network included three miRNAs, seven mRNAs and four lncRNAs (Figure 10).

### Correlation between DEGs and differential immune cells in synovial tissues following knee trauma

**FIGURE 11:**
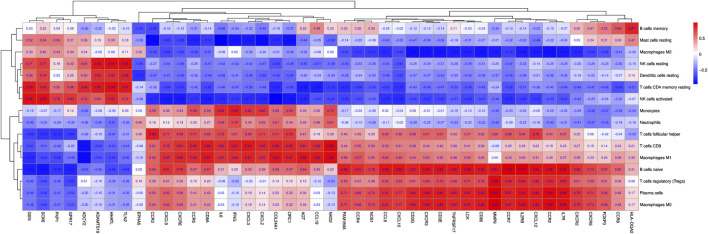
The correlation heat map graph is shown by the rate of correlation between hub genes and immune cells.

Forty-five immune/inflammatory-related hub genes from the PPI and ceRNA networks were selected mainly based on the degrees of the hub genes. CD8 T cells and M1 macrophages were significantly increased in inflamed synovial tissues. Correlations among the 45 DEGs and 16 kinds of immune cells were analysed between inflamed and normal synovial tissues in knee trauma, as shown in the correlation heatmap graph (Figure 11). Significantly related DEGs and immune cells were screened by |R| > 0.40 and *p* < 0.001. The results indicated that MMP9 correlated positively with M0 macrophages (R = 0.99, *p* = 0.00033) and plasma cells (R = 0.99, *p* = 0.00033), NKD2 correlated positively with CD8 T cells (R = 0.98, *p* = 0.00090), and CCR7 (R = 0.99, *p* = 0.00003) and IL2RB (R = 0.99, *p* = 0.00017) correlated positively with naive B cells.).

## Discussion

ACL and meniscus tears are common knee injuries. Emerging evidence suggests that joint trauma is a major cause of OA. Moreover, approximately 50% of patients with knee trauma eventually develop PTOA several years after their injuries (Oiestad et al., 2009; Stiebel et al., 2014). Nonetheless, the factors that contribute to PTOA development remain unclear. Recently, attention has turned to the importance of synovial inflammation, which may play a role in OA progression (Sellam and Berenbaum, 2010). To better explore the etiology and evolution of PTOA, the specific molecular mechanisms of synovial inflammation following knee injuries need to be understood. Noncoding RNAs have important regulatory roles in the pathogenesis of OA (Reynard and Barter, 2020; Ali et al., 2021). By regulating the expression of specific gene targets, noncoding RNAs may play an essential role in establishing and maintaining the homeostatic balance of biological systems. Furthermore, studies have also suggested that biological changes in the joint after injury may contribute to PTOA development (Amano et al., 2018; Clair et al., 2019). There are some previous studies on synovial inflammation, but the overall design of these studies involved RNA-seq for synovial tissues from inflammatory/degenerative joint diseases compared with healthy individuals/trauma patients (Woetzel et al., 2014; Lauwerys et al., 2015). To our knowledge, high-throughput RNA sequencing of inflamed and normal synovial tissues following ACL/meniscus injury have not been performed. Thus, in the present study, we conducted RNA-seq to systematically analyze the differentially expressed lncRNAs, miRNAs and mRNAs between inflamed and normal synovial membranes and established a ceRNA regulatory network to uncover the corresponding underlying pathogenesis of PTOA.

In the present study, 2793 mRNAs, 211 miRNAs and 3392 lncRNAs were significantly upregulated or downregulated between inflamed and normal synovial membranes of patients following knee trauma. To further investigate the potential biological roles of the aberrantly expressed mRNAs, GO and KEGG pathway analyses. The top significantly enriched upregulated GO terms were conducted in the BP category in terms of fold enrichment, such as immune response (GO: 0006955), immune system process (GO: 0002376), inflammatory response (GO: 0006954), cytokine-mediated signaling pathway (GO: 0019221), and chemotaxis (GO: 0006935), revealed the occurrence of a localized synovial membrane immune response and inflammatory process in a subset of knees with ACL/meniscus injury. Previous studies have revealed that subjects diagnosed with primary OA exhibit elevated percentages of activated macrophages and T cells in peripheral blood, synovial fluid, and synovial tissues (Li et al., 2017; Rosshirt et al., 2019). Specifically, a recent study has revealed a T-cell-predominant immune profile in the synovial fluid following ACL and meniscus injuries (Kim-Wang et al., 2021). The immune cells present after joint injuries may play a vital role in the development of PTOA. Further studies will be needed to investigate the relationships among macrophages, monocytes, and T cells to better understand the pathogenesis of PTOA following joint injury.

The pathway analyses revealed 41 upregulated and 1 downregulated enriched pathways, which helped to further elucidate the underlying functions of the differentially expressed mRNAs. The notable upregulated pathway terms included the systemic lupus erythematosus, hematopoietic cell lineage, Th17-cell differentiation, Th1- and Th2-cell differentiation, cytokine–cytokine receptor interaction, NF-kappa B signaling pathway, and RA signaling pathway terms. Several signaling pathways, such as the hematopoietic cell lineage pathway, the phagosome pathway, extracellular matrix receptor interaction, natural killer cell–mediated toxicity, and T cell receptor signaling pathways, which are similar to the results of a synovial fluid proteomic study (King et al., 2020), might be associated with the inflammatory response after joint injury. Moreover, eight of the top 10 upregulated pathways in KEGG pathway analysis were related to immune diseases or the immune response. The upregulation of the RA pathway, which has also been reported in the knee synovium in the context of OA (Xiang et al., 2019a), suggests that the initial synovial membrane response to ACL/meniscus injuries is similar to RA (King et al., 2020). The cardinal sign of RA is damage to cartilage and bone owing to synovial invasion into adjacent articular structures (Smolen et al., 2018). Therefore, a RA-like synovial response after ACL/meniscus injuries may imply progressive cartilage degradation (Alonso et al., 2020). In addition, Th1-, Th2- and Th17-cell differentiation pathways were significantly upregulated in this study. Similarly, studies have revealed that high percentages of Th cells are present in blood samples and synovial fluid from RA and OA subjects (Lurati et al., 2015; Rosshirt et al., 2019). Recently, Kim-Wang et al. (2021) also found that the numbers of immune cells, primarily T cells with multiple Th phenotypes, are elevated in the synovial fluid following ACL and meniscus injuries, while the numbers of Th1, Th2, and Th17 cells are the dominant populations of the CD4 subsets. Furthermore, T17 cells can produce IL-17, which causes synovial fibroblasts, chondrocytes, macrophages, and osteoclasts to elicit a cascade and finally promotes inflammation, cartilage degradation, and changes in bone metabolism (Koenders et al., 2006). Therefore, these enriched immune cells may play an important role in joint changes that account for the pathogenesis of PTOA following ACL and meniscus injuries.

Moreover, we conducted PPI network analysis to further characterize the differentially expressed mRNAs. EPHA5 and TLN2 were involved in the network; these findings were verified by PCR analyses. EphA5, a member of the Eph-Ephrin signaling axis, can play a dual role in the growth regulation of human bone marrow stromal cells, which might be involved in bone remodeling (Yamada et al., 2016; Arthur and Gronthos, 2021). Another study has demonstrated that miR-34a can target EphA5, resulting in negative modulation of chondrogenesis (Kim et al., 2011). In this study, EphA5 was downregulated in the inflamed synovium after knee injury, which might be related to the progression of PTOA.

Analyses of ceRNA networks, noncoding endogenous transcripts that compete for shared miRNAs, have exciting implications for diverse biological systems and pathophysiological conditions (Tay et al., 2014). Because miRNAs are the cores of ceRNA networks, the DE miRNAs in RNA-seq analysis were firstly evaluated by a secondary PCR analysis approach. Our results revealed that four miRNAs were DE in inflamed synovium compared with normal synovium. Previous studies have shown that miR-486-5p is upregulated in patients with knee OA and that it might aggravate the progression of OA (Kong et al., 2017; Shi et al., 2018). Inhibition of miR-486-5p significantly increases cell proliferation and decreases apoptosis in murine chondrogenic cells (Chang et al., 2020). However, Chen et al. reported that exosomal miR-486-5p derived from RA fibroblast-like synoviocytes can promote osteoblast differentiation and alleviate the disease severity of RA (Chen et al., 2020). In our study, miR-486-5p was upregulated after knee injury. Therefore, more in-depth studies are needed to investigate the biological role of miR-486-5p after knee trauma. Previous studies have also found that miR-199a-3p can directly regulate cyclooxygenase-2 expression and prostaglandin E2 production in IL-1β-stimulated human OA chondrocytes, suggesting that miR-199a-3p might be a novel target for OA therapy (Akhtar and Haqqi, 2012; Rasheed et al., 2016). Similarly, in our data, the expression of miR-199a-3p was downregulated in inflamed synovium, possibly indicating a destructive effect in articular cartilage.

Next, ceRNA networks were constructed using bioinformatics methods based on the differentially expressed mRNAs, lncRNAs and verified miRNAs. Then, subsets of RNAs in the networks were analyzed by PCR to establish a subnetwork. Recently, some mRNAs have been confirmed to be associated with OA, chondrogenesis or osteogenesis. For example, studies have revealed that bone marrow stromal cells and adipose stem cells electroporation-mediated transfer of a trio of SOX genes (SOX-5, SOX-6, and SOX-9) can enhance chondrogenesis potential *in vitro* and regeneration of defective cartilage (Im and Kim, 2011; Kim and Im, 2011). The levels of GSN, which was downregulated in our study, have also been shown to be decreased in patients with RA, suggesting local consumption of potentially anti-inflammatory proteins in the inflamed joint (Osborn et al., 2008). NKD2, a signal-inducible feedback antagonist of the canonical Wnt signaling pathway, can promote the differentiation of dental follicle stem/progenitor cells into osteoblasts (Chen et al., 2018).

With respect to lncRNAs in the ceRNA subnetwork, it has recently been reported that MIR4435-2HG is downregulated in OA and can regulate chondrocyte proliferation and apoptosis (Xiao et al., 2019). Moreover, further study has demonstrated that MIR4435-2HG can significantly suppress the progression of OA *via* the miR-510-3p/IL-17A axis (Liu et al., 2020). In the present study, MIR4435-2HG was upregulated in the inflamed synovium group after knee injury, which did not support our hypothesis that greater inflammation would lead to more cartilage breakdown. A recent study has also demonstrated that the concentrations of inflammatory markers in the synovial fluid are not associated with worse cartilage outcomes (Amano et al., 2018), which may be attributable to time-dependent effects on the levels of inflammatory markers or individual variations in susceptibility of the cartilage to inflammation. TMEM92-AS1 has been shown to promote gastric cancer progression by targeting CCL5, and further study has shown that it may affect leukocytes *via* regulation of the expression of granulocyte colony-stimulating factor in gastric cancer tissues (Song et al., 2021). It is likely that deregulation of TNXA plays a key role in the development and/or progression of bladder cancer (Zhu et al., 2014). Cerox1 has been demonstrated to regulate mitochondrial oxidative phosphorylation to decrease reactive oxygen species production by binding to miR-488-3p (Sirey et al., 2019). Surprisingly, most of the above lncRNAs have not been extensively investigated in the context of OA. Thus, further studies are needed to assess their characteristics.

GO and KEGG enrichment analyses revealed that immune cells infiltrated the inflamed synovial tissues after ACL and meniscus injuries. Increasing evidence has shown that immune cell infiltration plays a key role in synovial inflammation and joint damage (Zhou et al., 2021; Hu et al., 2022). In this study, CD8 T cells and M1 macrophages were significantly increased in inflamed synovial tissues. Therefore, it is essential to further investigate the correlation among the important hub genes and immune cells following knee trauma. In this study, MMP9 correlated positively with M0 macrophages. M0 macrophages differentiate into M1 and M2 macrophages under specific circumstances, which may play a role in the immune imbalance related to RA pathogenesis. A study of esophageal cancer showed that MMP-9 might regulate tumour-associated macrophages (Yuan et al., 2021). Previous studies have shown that MMP-9 and macrophages participate in the pathogenesis of RA (Xu et al., 2021) and OA (Ostojic et al., 2021). Our results indicate that MMP9 is significantly overexpressed. Therefore, we speculate that MMP9 may participate in the occurrence and development of PTOA through the MMP9-macrophage axis, which needs to be verified by further experiments. In the present study, NKD2 correlated positively with CD8 T cells. Recently, researchers observed a strong correlation between NKD2 expression and pro-inflammatory cytokine production in effector T cells, demonstrating that NKD2 might regulate the function of effector T cells, especially in an inflammatory status (Wu et al., 2022). Intriguingly, the biological function of the NKD2-CD8 T-cell axis merits further investigation to understand the underlying mechanism and provide potential therapeutic targets for PTOA.

In summary, the present study conducted RNA-seq between inflamed and normal synovial tissues, and bioinformatics/PCR experiments were used to analyse the transcriptional expression characteristics of knee ACL/meniscus injuries. The most predominant features of synovitis after knee trauma were an increased immune response and immune cell infiltration. The essential molecules of the regulatory network identified in this study provide potential biomarkers for PTOA diagnosis, development and treatment. However, subsequent studies investigating the relationship between immune cells/essential molecules and joint degeneration are needed.

The small sample size is an obvious limitation of this study. Three patients with an inflamed synovium and 3 patients with a normal synovium do not fully reflect all the aspects of synovial profiling. Thus, it is necessary to expand the sample size to reduce these limitations. In addition, the functions of the above lncRNAs, miRNAs and mRNAs in ceRNA networks have not been explored in various cells related to PTOA. Further fundamental studies are needed to investigate the roles of these molecules in the pathogenesis and development of PTOA.

## Conclusion

In conclusion, our study is the first to detect and analyze mRNA, lncRNA, and miRNA differences between inflamed and normal synovial membranes in injured ACL and/or meniscus. This study reveals novel ceRNA and PPI networks, which may be valuable for PTOA pathogenesis and diagnosis. These immune cells that are present following ACL and meniscus injuries may contribute to the development of PTOA. In addition, this study shows that immune-related DEGs have the potential to serve as biomarkers for PTOA treatment. Future studies that investigating the relationship between immune cells/essential molecules and joint degeneration may contribute to a profound understanding of the pathophysiology of PTOA after joint injury.

## Data Availability

The datasets presented in this study can be found in online repositories. The name of the repository and accession numbers can be found below: GEO, NCBI; GSE213070.

## Supplementary material

SUPPLEMENTARY TABLE S1 The miRNA primers used in qRT-PCR.

SUPPLEMENTARY TABLE S2 The mRNA primers used in qRT-PCR.

SUPPLEMENTARY TABLE S3 The lnc RNA primers used in qRT-PCR.

SUPPLEMENTARY DATA S1 Differentially expressed miRNAs between the inflamed group and the control group.

SUPPLEMENTARY DATA S2 Differentially expressed mRNAs between the inflamed group and the control group.

SUPPLEMENTARY DATA S3 Differentially expressed lncRNAs between the inflamed group and the control group.

SUPPLEMENTARY DATA S4 Upregulated GO Biological Process analysis between the two groups.

SUPPLEMENTARY DATA S5 Downregulated GO Biological Process Analysis between the two groups.

SUPPLEMENTARY DATA S6 Upregulated KEGG Pathway Analysis between the two groups.

SUPPLEMENTARY DATA S7 Downregulated KEGG Pathway Analysis between the two groups.

SUPPLEMENTARY DATA S8 Overlapping genes with two immune databases.

SUPPLEMENTARY DATA S9 The topological characteristics of nodes in the PPI networks.

SUPPLEMENTARY DATA S10 The topological characteristics of nodes in the ceRNA networks.

## Glossary

OA: Osteoarthritis
ACL: Anterior cruciate ligament
PTOA: Posttraumatic osteoarthritis
RNA-seq: RNA sequencing
miRNAs: MicroRNAs
lncRNAs: Long noncoding RNAs
ceRNA: Competing endogenous RNA
H&E: Hematoxylin-eosin
RT–PCR: Real-time polymerase chain reaction
DE: Differentially expressed
GO: Gene Ontology
KEGG: Kyoto Encyclopedia of Genes and Genomes
PPI: Protein–protein interaction
BP: Biological process
RA: Rheumatoid arthritis
IL6: Interleukin 6
IL2: Interleukin 2
IL17: Interleukin 17
IL-17A: Interleukin 17A
IL-1: Interleukin 1
CCR7: Cinnamoyl-CoA reductase 7
CCR5: Cinnamoyl-CoA reductase 5
CCR3: Cinnamoyl-CoA reductase 3
CCR2: Cinnamoyl-CoA reductase 2
CCR8: Cinnamoyl-CoA reductase 8
C3: Complement C3
CXCR3: C-X-C motif chemokine receptor 3
CXCL8: C-X-C motif chemokine ligand 8
CXCL1: C-X-C motif chemokine ligand 1
CXCL10: C-X-C motif chemokine ligand 10
CXCL2: C-X-C motif chemokine ligand 2
CCL5: C-C motif chemokine ligand 5
AGT: Angiotensinogen
NOS1: Nitric oxide synthase 1
GSN: Gelsolin
ORC1: Origin recognition complex subunit 1
SOX: Sex-determining region Y-box transcription factor
SOX-5: Sex-determining region Y-box transcription factor 5
SOX-6: Sex-determining region Y-box transcription factor 6
SOX-9: Sex-determining region Y-box transcription factor 9
NKD2: Naked cuticle homolog 2
ADAMTS19: A disintegrin and metalloproteinase with thrombospondin type 1 motif 19
EPHA5: Ephrin receptor A5
TLN2: Talin 2
TNXA: Tenascin-XA
CEROX1: cytoplasmic endogenous regulator of oxidative phosphorylation 1
Th1: Helper T1
Th2: Helper T2
Th17: Helper T17
DEGs: Differentially expressed genes
MMP9: Matrix metallopeptidase 9
IL2RB: Interleukin 2 receptor subunit beta
RA: Rheumatoid arthritis
