# Supplementary material for: Construction and analysis of a lncRNA–miRNA–mRNA competing endogenous RNA network from inflamed and normal synovial tissues after anterior cruciate ligament and/or meniscus injuries
Source: Front Genet. 2022 Oct 17;13:983020. doi: 10.3389/fgene.2022.983020 (PMC9619217; doi:10.3389/fgene.2022.983020)
Supplement: Supplementary file 1 [file DataSheet1.ZIP › additional files 6.27/Additional file 1 Table S3.docx]

### Table S3 The lnc RNA primers used in qRT-PCR.

| **Primer name** | **Sequence (5′-3′)** |
| --- | --- |
| Hu-GAPDH-F | ACAACTTTGGTATCGTGGAAGG |
| Hu-GAPDH-R | GCCATCACGCCACAGTTTC |
| Hu-STAG3L1-F | ACCAGGAGAGATTGCTTGCTT |
| Hu-STAG3L1-R | ATTAGCCACCTTCCAACTCCAA |
| Hu-VIM-AS1-F | CCTGGCTTTTACAAACTGC |
| Hu-VIM-AS1-R | AAATGGGGAAACTGGTGA |
| Hu-miR-4435-2HG-F | AATTTGCCACCACCCTGTGA |
| Hu-miR-4435-2HG-R | ATGCCGTTTTAGGGGGACAG |
| Hu-TMEM92-AS1-F | GTCTCGGCGGGAGATTAAGT |
| Hu-TMEM92-AS1-R | AGGTCTGTCTTGGGGTCTCC |
| Hu-TNXA-F | CCCAGTACGACTCCTTCCA |
| Hu-TNXA-R | TCACGGGCAGAGAAGACA |
| Hu-CEROX1-F | GTCCAGACATCGCCCACTC |
| Hu-CEROX1-R | TGCTCGCAGACCTAATGCAA |
| Hu-DUSP5P1-F | TCATCAGCCAGTGTGGAA |
| Hu-DUSP5P1-R | CCAAGGTAGAGGGAGGAAA |
| Hu-LINC00299-F | AACTTGAGAGGGGTGGGT |
| Hu-LINC00299-R | TCAGGCACAGAGTCCAGAT |
