# Supplementary material for: Construction and analysis of a lncRNA–miRNA–mRNA competing endogenous RNA network from inflamed and normal synovial tissues after anterior cruciate ligament and/or meniscus injuries
Source: Front Genet. 2022 Oct 17;13:983020. doi: 10.3389/fgene.2022.983020 (PMC9619217; doi:10.3389/fgene.2022.983020)
Supplement: Supplementary file 1 [file DataSheet1.ZIP › additional files 6.27/Additional file 1 Table S2.docx]

### Table S2 The mRNA primers used in qRT-PCR.

| **Primer name** | **Sequence (5′-3′)** |
| --- | --- |
| Hu-GAPDH-F | ACAACTTTGGTATCGTGGAAGG |
| Hu-GAPDH-R | GCCATCACGCCACAGTTTC |
| Hu-NOS1-F | TCCAGCAATAGCATCCAAG |
| Hu-NOS1-R | GACAAGTGACCCATCCAAA |
| Hu-SPIB-F | CGAGGGGAAGGGATCCGA |
| Hu-SPIB-R | GGAGGAGAACTGGAAGACGC |
| Hu-COL24A1-F | TGCAAGGTGATGTTGGAC |
| Hu-COL24A1-R | TGGTTCCCCAGTTCCTC |
| Hu-PLA2G2A-F | TGCCACCTGTTTTGCTAGA |
| Hu-PLA2G2A-R | GGGAGGGAGGGTATGAGA |
| Hu-GSN-F | CCAGCGAGGCAGAGAAG |
| Hu-GSN-R | GTGGGGATGTGCGGTAG |
| Hu-EMP1-F | GCTGGCTGGTATCTTTGTG |
| Hu-EMP1-R | TGTCTTGAGGGCATCTTCA |
| Hu-TLN2-F | GCTGGAGTTGGACATGGT |
| Hu-TLN2-R | GCTTACACACGGAGGCA |
| Hu-NKD2-F | TCCGACAGCAAACAGCAAC |
| Hu-NKD2-R | CGAGGGCAGAAGAGCAGA |
| Hu-ORC1-F | GTTGTTCCACCGAGATTCA |
| Hu-ORC1-R | CGAGCACGTTTCTTAGGAG |
| Hu-SOX6-F | ACGCCTGGAAGCATTTC |
| Hu-SOX6-R | TCAGGGGCATACCTGTTTA |
| Hu-EPHA5-F | GCACATTTTCAGCAGGATG |
| Hu-EPHA5-R | GGGAATGGTTACAACGGAT |
| Hu-COL6A6-F | TTCGTGGAGACTTTTGGAG |
| Hu-COL6A6-R | TTGAACAACAGATGCCAGA |
| Hu-PCDH8-F | GGCGACGAGAACGACAA |
| Hu-PCDH8-R | CGCACCGACACCTCATAG |
| Hu-ADAMTS19-F | GTGGTGTAAGGCTGGAGAA |
| Hu-ADAMTS19-R | CTCTCGACTGCTGATCCC |
