# Supplementary material for: Construction and analysis of a lncRNA–miRNA–mRNA competing endogenous RNA network from inflamed and normal synovial tissues after anterior cruciate ligament and/or meniscus injuries
Source: Front Genet. 2022 Oct 17;13:983020. doi: 10.3389/fgene.2022.983020 (PMC9619217; doi:10.3389/fgene.2022.983020)
Supplement: Supplementary file 1 [file DataSheet1.ZIP › additional files 6.27/Additional file 1 Table S1.docx]

### Table S1 The miRNA primers used in qRT-PCR.

| **Primer name** | **Sequence (5′-3′)** |
| --- | --- |
| U6-F | CTCGCTTCGGCAGCACA |
| U6-R | AAACGCTTCACGAATTTGCGT |
| Hu-miR-142-5p-RT | CTCAACTGGTGTCGTGGAGTCGGCAATTCAGTTGAGAGTAGT |
| Hu-miR-142-5p-F | ACACTCCAGCTGGGCATAAAGTAGAAAGC |
| Hu-miR-199a-3p-RT | CTCAACTGGTGTCGTGGAGTCGGCAATTCAGTTGAGTAACCA |
| Hu-miR-199a-3p-F | ACACTCCAGCTGGGACAGTAGTCTGCACAT |
| Hu-miR-423-5p-RT | CTCAACTGGTGTCGTGGAGTCGGCAATTCAGTTGAGTGAGGG |
| Hu-miR-423-5p-F | ACACTCCAGCTGGGAAAAGCTCGGTCTGAGGC |
| Hu-miR-31-3p-RT | CTCAACTGGTGTCGTGGAGTCGGCAATTCAGTTGAGATGGCA |
| Hu-miR-31-3p-F | ACACTCCAGCTGGGTGCTATGCCAACATAT |
| Hu-miR-144-3p-RT | CTCAACTGGTGTCGTGGAGTCGGCAATTCAGTTGAGAGTACA |
| Hu-miR-144-3p-F | ACACTCCAGCTGGGTACAGTATAGATGA |
| Hu-miR-486-5p-RT | CTCAACTGGTGTCGTGGAGTCGGCAATTCAGTTGAGCTCGGG |
| Hu-miR-486-5p-F | ACACTCCAGCTGGGTCCTGTACTGAGCTGC |
| Hu-miR-365a-3p-RT | CTCAACTGGTGTCGTGGAGTCGGCAATTCAGTTGAGATAAGG |
| Hu-miR-365a-3p-F | ACACTCCAGCTGGGTAATGCCCCTAAAAAT |
| Hu-miR-3184-3p-RT | CTCAACTGGTGTCGTGGAGTCGGCAATTCAGTTGAGTGAGGG |
| Hu-miR-3184-3p-F | ACACTCCAGCTGGGAAAGTCTCGCTCTCTGC |
| Hu-miR-103a-3p-RT | CTCAACTGGTGTCGTGGAGTCGGCAATTCAGTTGAGTCATAG |
| Hu-miR-103a-3p-F | ACACTCCAGCTGGGAGCAGCATTGTACAGGG |
| Hu-miR-103b-RT | CTCAACTGGTGTCGTGGAGTCGGCAATTCAGTTGAGGGCTTC |
| Hu-miR-103b-F | ACACTCCAGCTGGGCAAGGCAGCACTGTAAA |
| Hu-miR-21-3p-RT | CTCAACTGGTGTCGTGGAGTCGGCAATTCAGTTGAGACAGCC |
| Hu-miR-21-3p-F | ACACTCCAGCTGGGCAACACCAGTCGATG |
| miR-R | TGGTGTCGTGGAGTCG |
